# Toward Eliminating Discontinuous Yielding Behavior of the EA4T Steel

**DOI:** 10.3390/ma14206121

**Published:** 2021-10-15

**Authors:** Jian-Zhi Chen, Qin Du, Guang-Ping Zhang, Bin Zhang

**Affiliations:** 1 Key Laboratory for Anisotropy and Texture of Materials (Ministry of Education), School of Materials Science and Engineering, Northeastern University, 3-11 Wenhua Road, Shenyang 110819, China; jzchen0907@163.com; 2 Beijing North Vehicle Group Co. Ltd., 5 Zhujiafenwuli, Beijing 100072, China; duqin12345@sina.com; 3 Shenyang National Laboratory for Materials Science, Institute of Metal Research, Chinese Academy of Sciences, 72 Wenhua Road, Shenyang 110016, China

**Keywords:** steel, tensile deformation, discontinuous yielding, carbide, heat treatment

## Abstract

Cold-rolled EA4T steel was heat-treated by inter-critical holding at 755 °C for 90, 120, 180, and 240 s, respectively, and then quenching in water. The tensile testing results of the EA4T specimens show an evident transition from the discontinuous yielding to the continuous yielding of the steel specimens by prolonging the holding time. A novel relationship between the discontinuous yielding behavior of tensile-deformed steel specimens and the carbide size was proposed based on experimental results and Cottrell’s theory. The model may provide a new clue for avoiding discontinuous yielding and improving mechanical properties of metals with static strain aging behaviors.

## 1. Introduction

Discontinuous yielding (DY), a common behavior occurring in low-carbon steels subjected to tensile deformation, refers to the interaction between the solute atom and the mobile dislocation [1,2,3,4]. For the DY, the appearance of yield point elongation (YPE) may be beneficial to resisting strain localization [5,6] and thus improves the strength–ductility combination [5,7]. However, the DY behavior can also roughen the product surface [6,8,9], which should be avoided during the thermal processing of metal parts. To avoid the DY of metals, many strategies have been proposed. Among these strategies, a simple and common method is to apply a small pre-strain to the metal before the thermal processing. However, this method is not appropriate for all metals [8], and the pre-strained metals may exhibit the DY again after aging at room temperature for a certain time [10]. In addition, tailoring the heat-treatment process is an effective way to eliminate DY behavior. Effects of carbon concentration [2,8,9], phase content [8,11], grain size [12,13] and other microstructure factors [5,11,14,15,16] on the DY behavior of metals have been investigated for recent years. An increase of holding time in the heat-treatment process can promote carbide precipitation [17,18] and reduce solute carbon concentration in the matrix, beneficial to prohibiting the DY behavior. However, carbide coarsening and grain growth also occur with increasing the holding time [19,20,21], and thus degrade the strength of metals. Thus, the optimum holding time for avoiding the DY behavior and keeping superior mechanical properties is a key factor.

EA4T steel (25CrMo4) is a kind of standardized steel used to produce high-speed railway axles, which are required to possess a good combination of strength and ductility [22,23]. To obtain finer microstructures and superior mechanical properties, thermal processes such as hot forging are often applied in the manufacturing process of the EA4T axle [24]. Thus, to eliminate the DY phenomena and optimize mechanical properties of EA4T steel, a novel model related to the carbide size and the holding time based on Cottrell’s theory was proposed.

## 2. Materials and Methods

Table 1 presents nominal chemical compositions (in wt. %) of the EA4T steel studied here. The as-received EA4T steel with tempered sorbite was first held at 910 °C for 60 min and quenched in water to room temperature (RT), followed by subsequent tempering at 250 °C for 180 min and air cooling. Then, four steel plates with dimensions of 50 mm × 15 mm × 15 mm cut from the heat-treated EA4T steel plates were cold-rolled with a reduction in the thickness of 80% (*ε* = 186%). Finally, in order to better study the DY phenomena and optimize the mechanical properties, the cold-rolled plates were inter-critical held at 755 °C for different holding times (*t*) of 90, 120, 180, and 240 s, followed by water quenching to RT based on the A_1_ and A_3_ temperatures (~729 °C and ~811 °C) of EA4T steel [24] and the theoretical formula of heat-treatment holding time [25].

Microstructure observations on the rolling direction (RD)-normal direction (ND) section of specimens were carried out using a scanning electron microscope (SEM, Leo Supra 35, 20 kV, Zeiss Co., Oberkochen, Germany) and a transmission electron microscope (TEM, JEM-2100F, 200 kV, JEOL Co., Tokyo, Japan). Sizes of carbide and martensite were evaluated based on TEM and SEM images. Three dog-bone-shaped specimens with gauge dimensions of 3 mm × 1 mm × 0.25 mm for each heat-treatment condition were prepared from the steel plates, and then ground mechanically and electro-polished. Uniaxial tensile tests along the RD of specimens were carried out on an INSTRON 5848 Microtester (Instron Co., Norwood, MA, USA) with a strain rate of 1 × 10^−3^ s^−1^ at RT, and the tensile strain was measured by a non-contact laser extensometer (MTS LX 300, MTS Co., Minneapolis, MN, USA).

## 3. Results and Discussion

**Figure 1:**
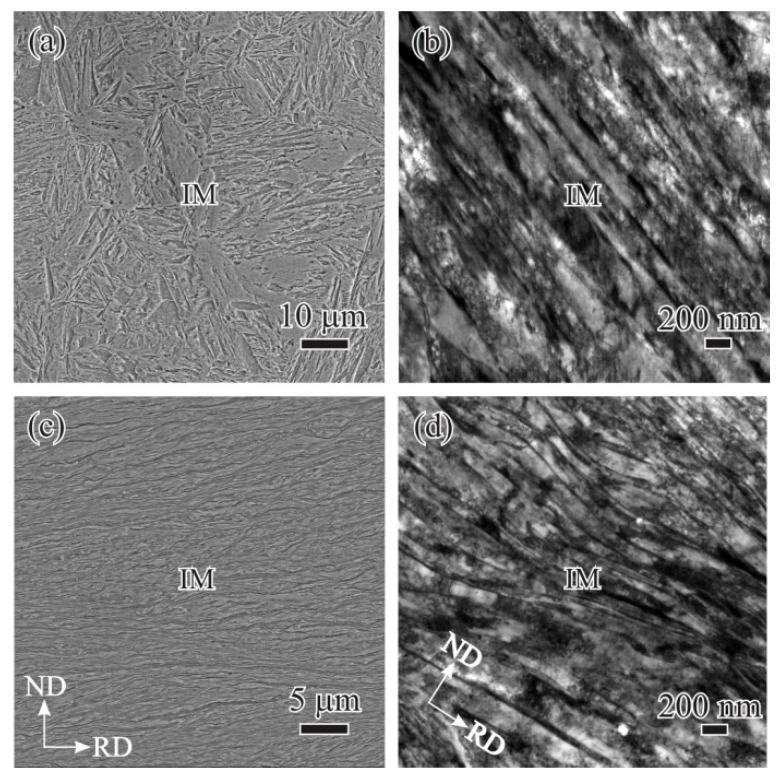
SEM and corresponding TEM images of tempered martensite (**a**,**b**) before and (**c**,**d**) after the cold-rolling.

Figure 1a,b show SEM and TEM images of the microstructure after 910 °C quenching and 250 °C tempering processes, respectively. The microstructure is mainly composed of lath-like tempered initial martensite (IM). After the 80% reduction of the cold-rolling process, the severely deformed IM preferentially distributed along the RD, as shown in Figure 1c,d.

**Figure 2:**
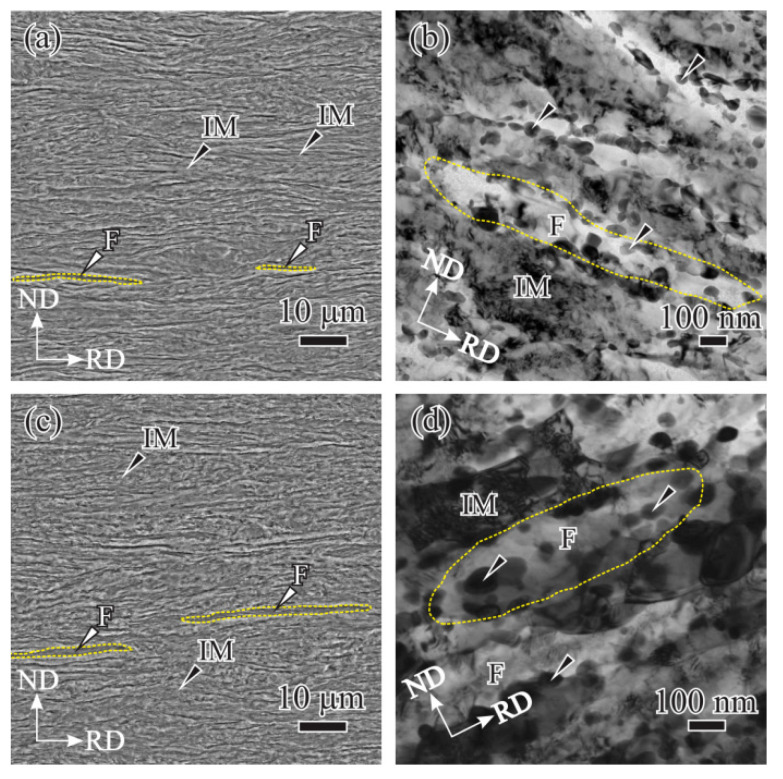
SEM and corresponding TEM images of quenched microstructure after inter-critical holding at 755 °C for (**a**,**b**) 90 s and (**c**,**d**) 120 s.

After holding at 755 °C for *t* = 90 s and then quenching, the microstructure composed of lamella ferrite regions and IM was obtained, as pointed by white and black arrows in Figure 2a. Ferrite regions contain several lamella ferrite grains parallel to the RD, as circled by the yellow dotted line in Figure 2b. Apart from the lamella ferrite, carbides precipitating at ferrite boundaries could be also observed, as indicated by black arrows shown in Figure 2b. As *t* = 120 s, lamella ferrite regions and IM remained (Figure 2c), but the ferrite grain in *t* = 120 s specimens is wider than that of *t* = 90 s specimens by comparing Figure 2b,d. Moreover, the boundaries between the ferrite and the IM are hard to be distinguished, and carbides could be observed inside the ferrite apart from ferrite boundaries, as indicated by the black arrows in Figure 2d.

**Figure 3:**
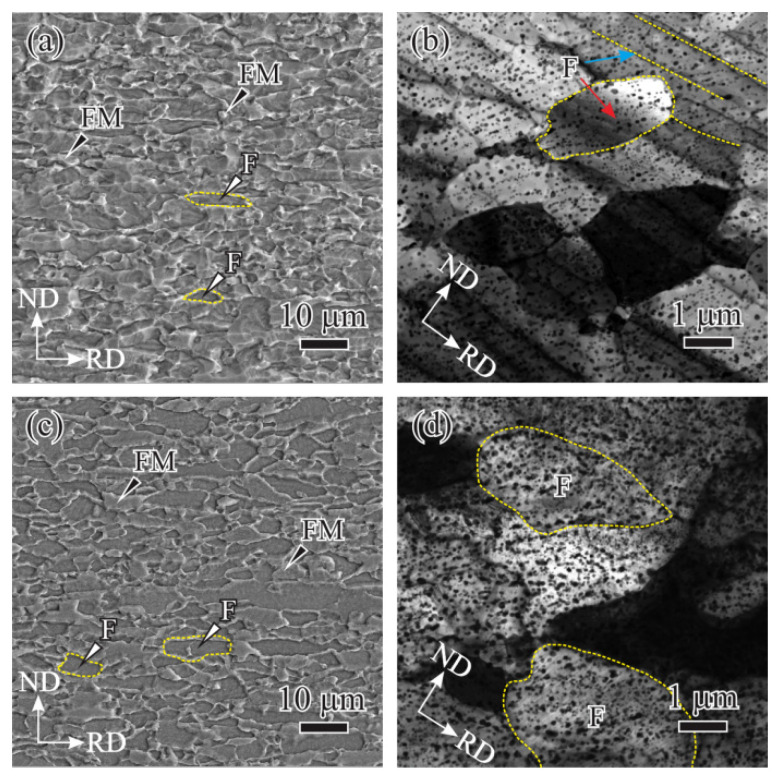
SEM and corresponding TEM images of quenched microstructure after inter-critical holding at 755 °C for (**a**,**b**) 180 s and (**c**,**d**) 240 s.

As *t* = 180 s, IM was exhausted and replaced by the fresh martensite (FM), the quenching product of austenite generated in the inter-critical holding, the obtained microstructure is consisted of ferrite and FM, as indicated by white and black arrows in Figure 3a. The equiaxed and the lamellar ferrites were observed in the *t* = 180 s specimen, as shown by red and blue arrows in Figure 3b. The increasing solid solution carbon released from the exhausted IM promoted the carbide precipitation in surrounding ferrite, thus the volume fraction of carbides (*V*_c_) increased with prolonging *t* (see Figure 2b,d and Figure 3b). As *t* further increased from 180 s to 240 s, the size (*d*_M_) and the volume fraction (*V*_M_) of FM also increased due to the austenite growth (see Figure 3a,c). In addition to *V*_c_, the increase of carbide size (*r*_c_) due to the Ostwald ripening was also found (see Figure 2b,d and Figure 3b,d).

**Figure 4:**
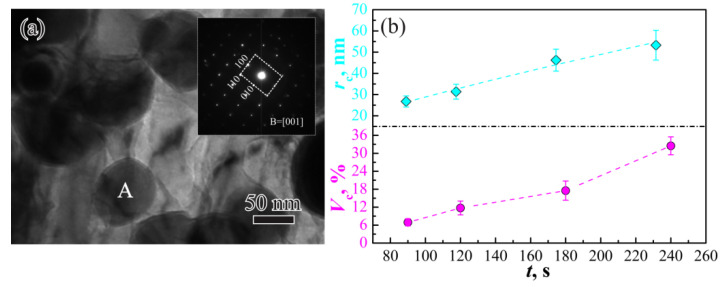
(**a**) TEM image and selected area electron diffraction of the carbide (Site A); (**b**) variations of *r*_c_ and *V*_c_ with *t*.

To reveal the carbide type, the selected area electron diffraction pattern (SAED) of carbide (site A in Figure 4a) along [1] zone axis was obtained (inset of Figure 4a), and the cementite crystal structure was determined. Both *r*_c_ and *V*_c_ estimated according to TEM images increase with prolonging *t* (Figure 4b). Continuous release of solute carbon from the IM happened in the inter-critical holding, enhancing the precipitation of carbide. Thus, *V*_c_ exhibits an increasing tendency with prolonging *t*, as indicated by a violet line in Figure 4b. Meanwhile, the Ostwald ripening process driven by the reduction of interfacial energy happened after the carbide precipitation [26,27,28], and *r*_c_ also increases with increasing *t*, as indicated by cyan points in Figure 4b. Carbon is the controlling element in the Ostwald ripening process, and *r*_c_ is generally proportional to *t^n^* [29,30,31,32]. Thus, *n* was defined as 0.79 here based on the fitting relationship between *r*_c_ and *t* (cyan dotted line in Figure 4b),

(1) $$r_{c}=2.28+0.69\times t^{0.79}$$

In addition, according to the Gibbs-Thompson Equation [33,34], the equilibrium concentration of carbon (C_αrc_) in the matrix can be expressed by Equation (2) when the size of carbide in radius is r_c_,

(2) $$C_{{\alpha r}_{c}}\approx C_{\alpha 0}\cdot \left(1+\frac{2{\gamma V}_{m}}{C_{P}{\mathrm{RTr}}_{c}}\right)$$

Here, C_α0_ is the equilibrium concentration of carbon in the ferrite matrix when r_c_ is infinite. γ is the interfacial energy between the cementite and the matrix, V_m_ is the molar volume of cementite, *C*_P_ is the equilibrium molar concentration of carbon in cementite, *R* and T are the gas constant and the thermodynamic temperature, respectively. According to Equation (2), one can find that the increase of r_c_ will lead to the decrease of C_αrc_ when other parameters are determined.

**Figure 5:**
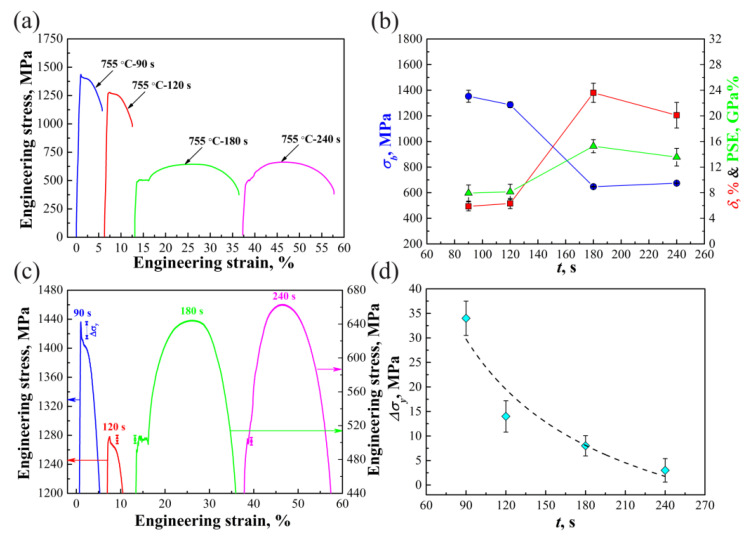
(**a**) Tensile engineering stress-strain curves; (**b**) variations of *σ*_b_, *δ* and PSE with *t*; (**c**) magnified upper parts of tensile curves; (**d**) relationship between Δ*σ*_y_ and *t*.

Figure 5a presents engineering stress-strain curves of different specimens, from which variations of tensile strength (*σ*_b_) and total elongation (*δ*) with increasing *t* were obtained, as shown in Figure 5b. As *t* is prolonged from 90 s to 180 s, *σ*_b_ decreases but *δ* increases. This is mainly caused by the increase of ferrite and the reduction of IM (see Figure 2a,c and Figure 3a). As *t* = 240 s, *σ*_b_ increases but *δ* decreases, as compared with that of *t* = 180 s specimens. This is mainly related to the increase of FM (see Figure 3a,c). The strength–ductility combination expressed by the product of strength and total elongation (PSE) is shown by the green line in Figure 5b, PSE of specimens increases first and then decreases with prolonging *t*, and the *t* = 180 s specimens exhibit the best strength–ductility combination. As *t* increases from 90 s to 180 s, PSE shows an increasing tendency, which is mainly related to the increase of ferrite and the recovery of strain hardening capability [5]. Further increasing *t* from 180 s to 240 s, the decrease of PSE happened due to the reduction of $\sqrt{V_{M}/d_{M}}$ [35,36], ~452 m^−1/2^ for *t* = 180 s specimens and ~435 m^−1/2^ for *t* = 240 s specimens. To better compare the DY behaviors of different specimens, the upper parts of tensile curves in Figure 5a are magnified, as shown in Figure 5c. For *t* = 90 s and 120 s specimens, the yield-drop phenomena occur, whereas no YPE appears. The disappearance of YPE is related to the limited volume fraction of ferrite and poor strain hardening capability [37,38,39]. As *t* = 180 s, YPE appears after the yield drop due to the increase of ferrite and the recovery of strain hardening capability. On the one hand, as *t* = 240 s, the increase of the ferrite size is not beneficial to increasing YPE [40]. On the other hand, the increase of *r*_c_ can lead to the reduction of carbon in ferrite (Equation (2)), which is also not good for enhancing YPE [8,14]. Hence, YPE of the *t* = 240 s specimen is lower than that of the *t* = 180 s specimen. According to the explanation of DY in the plastic deformation theory, dislocations are immobilized by solute atoms around them. Thus, to generate plastic strain, high stress is required to break dislocations away from the locking effects of the solutes, and therefore the yield drop indicates the immobilized dislocations have broken away from the solute atmosphere [41]. Here, yield-drop stress (Δ*σ*_y_) expressed by the gap between the upper yield point and the lower yield point (Δ*σ*_y_ = *σ*_upper_ − *σ*_lower_), as indicated by a double-headed arrow in Figure 5c, was defined to represent the extent of yield drop, one can find that Δ*σ*_y_ shows a decreasing trend with prolonging *t* (Figure 5d).

**Figure 6:**
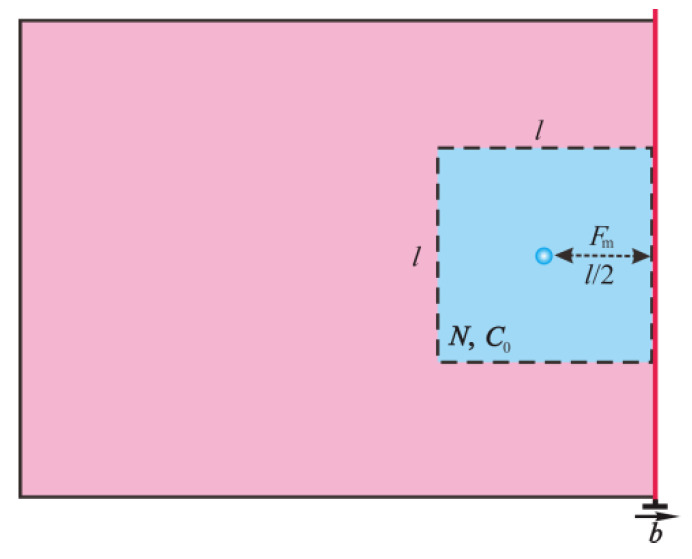
Schematic diagram of the interaction between the dislocation and the Cottrell atmosphere.

Generally, DY is related to the pinning effect of the Cottrell atmosphere on the mobile dislocation, and Δ*σ*_y_ in DY phenomena is affected by the carbon concentration [2]. The interaction force (*F*(r)) between the carbon atom and the edge dislocation can be expressed as [42],

(3) $$F\left(r\right)=-\frac{A\sin\theta }{r^{2}}$$

where *A* is the material constant. Here, the pinning force between Cottrell atmosphere and dislocation is approximately equal to that between carbon atoms in *l*^2^ square area (blue area shown in Figure 6) and edge dislocation (red line in Figure 6), *l*/2 is the mean distance between the carbon atoms and the edge dislocation. Then, according to Equation (3), the mean interaction force (*F*_m_) between carbon atoms in the blue area and the dislocation is equal to 4 *A*/*l*^2^ when *r* = *l*/2 and *θ* = −90°. Therefore, the stress (*σ*_c_) required for the edge dislocation to break away from the Cottrell atmosphere can be obtained [2],

(4) $$\sigma_{c}=F_{m}\cdot l^{2}C_{0}N\exp\left(\frac{l}{\frac{l}{2}}\right)\cdot \frac{1}{b}\approx A_{1}\cdot C_{0}$$

*C*_0_ is the equilibrium carbon concentration, *N* is the total number of atoms in unit volume, *b* is the Burgers vector, and *A*_1_ is a material constant. Let *C*_0_ = *C*_αrc_, Δ*σ*_y_ = *σ*_c_, one can obtain a model of Equation (5) by substituting Equation (2) into Equation (4),

(5) $$∆\sigma_{y}=A_{1}\cdot C_{\alpha 0}\cdot \left(1+\frac{2\gamma V_{m}}{C_{P}RTr_{c}}\right)=B+\frac{C}{r_{c}}$$

where both *B* and *C* are material constants. Substituting Equation (1) into Equation (5), the relationship between Δ*σ*_y_ and *t* in Figure 5d can be fitted as,

(6) $$∆\sigma_{y}=-24.43+\frac{1431.57}{r_{c}}=-24.43+\frac{1431.57}{2.28+0.69\times t^{0.79}}$$

**Figure 7:**
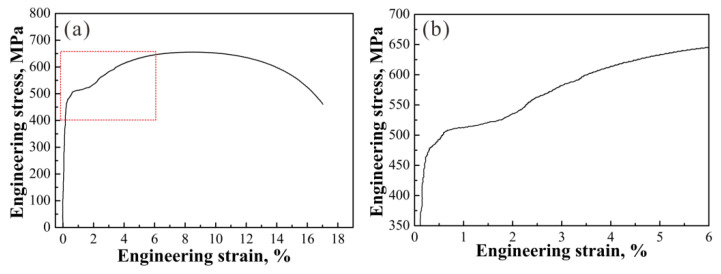
(**a**) Tensile engineering stress-strain curve and (**b**) magnified yielding zone of the specimen after inter-critical holding at 755 °C for 300 s and quenching.

Let Δ*σ*_y_ = 0 in Equation (6), then, *r*_c_ ≈ 59 nm and *t* ≈ 265 s are attained. Hence, to avoid the DY behavior, a condition with *r*_c_ ≥ 59 nm and *t* ≥ 265 s should be satisfied. Moreover, as shown in Figure 5b, the PSE values decrease when *t* > 180 s, indicating the deterioration of the strength–ductility combination. Therefore, to avoid DY behaviors and keep a better strength–ductility combination of the EA4T steel, one can obtain *r*_c_ ≈ 59 nm according to Equation (6), corresponding to *t* ≈ 265 s. To confirm the disappearance of discontinuous yielding at *t* ≥ 265 s, the tensile test of the specimen after inter-critical holding at 755 °C for 300 s and quenching was carried out. Figure 7a presents the tensile curve. *σ*_b_ and the *δ* are 656.59 MPa and 17.02% respectively, and the PSE is 11.18 GPa%, which is lower than *t* = 240 s specimens (Figure 5b). The yielding zone surrounded by the red rectangle in Figure 7a is magnified and shown in Figure 7b. One can find that the yield-drop phenomenon disappears, which confirms the reliability of the model.

Generally, the finer and more dispersed carbides are beneficial to improving mechanical properties of metals. However, for the metals with static strain aging behavior, the decrease of *r*_c_ will enhance Δ*σ*_y_, promoting undesirable DY behavior. A proper increase of *r*_c_ with prolonging *t* can help to change the tensile curve from the discontinuous yielding to the continuous yielding, thus, it is expected that the optimum *t* for the metals with continuous yielding and relatively superior mechanical properties can be achieved by tailoring *r*_c_ in the model of Equation (5).

## 4. Conclusions

In summary, by examining tensile stress-strain responses of the EA4T steel specimens subjected to inter-critical holding and quenching, an evident transition from the discontinuous yielding type to the continuous yielding type of tensile curves was observed with increasing holding time. A novel model related to the DY behavior was proposed based on the experimental results and Cottrell’s theory. The critical carbide size (*r*_c_ = 59 nm) and the holding time (*t* = 265 s) of the EA4T steel corresponding to continuous yielding and relatively superior strength–ductility combination were derived according to the model. It is expected that the model may be used to inhibit DY behaviors and improve the strength–ductility combination of other metals with static strain aging behaviors.

## Figures and Tables

**Table 1:** Main chemical compositions of the EA4T steel (wt. %).

C	Si	Mn	S	P	Cr	Cu	Mo	Ni	V
0.25	0.36	0.74	0.008	0.011	1.17	0.05	0.25	0.04	0.032

## Data Availability

Data sharing is not applicable for this article.
